# Dysregulation of antimicrobial peptide expression distinguishes Alzheimer’s disease from normal aging

**DOI:** 10.18632/aging.102650

**Published:** 2020-01-06

**Authors:** Min Wang, I-Feng Peng, Simin Li, Xianda Hu

**Affiliations:** 1 Department of Rheumatology, Peking Union Medical College Hospital, Chinese Academy of Medical Sciences and Peking Union Medical College, Beijing, China; 2 Suzhou Joekai Biotech LLC, Kunshan, China; 3 Department of Cariology, Endodontology and Periodontology, University of Leipzig, Leipzig, Germany; 4 Beijing Tibetan Hospital, China Tibetology Research Centre, Beijing, China

**Keywords:** Alzheimer's disease, antimicrobial peptide, innate immune system, *Drosophila melanogaster*, aging

## Abstract

Alzheimer's disease (AD) is an age-related neurodegenerative disease with unknown mechanism that is characterized by the aggregation of abnormal proteins and dysfunction of immune responses. In this study, an integrative approach employing *in silico* analysis and wet-lab experiment was conducted to estimate the degrees of innate immune system relevant gene expression, neurotoxic Aβ_42_ generation and neuronal apoptosis in normal *Drosophila melanogaster* and a transgenic model of AD. Results demonstrated mRNA levels of antimicrobial peptide (AMP) genes gradually increased with age in wild-type flies, while which exhibited a trend for an initial decrease followed by subsequent increase during aging in the AD group. Time series and correlation analysis illustrated indicated a potential relationship between variation in AMP expression and Aβ_42_ concentration. In conclusion, our study provides evidence for abnormal gene expression of AMPs in AD flies with age, which is distinct from the expression profiles in the normal aging process. Aberrant AMP expression may participate in the onset and development of AD by inducing or accelerating Aβ deposition. These findings suggest that AMPs may serve as potential diagnostic biomarkers and therapeutic targets. However, further studies are required to elucidate the pathological effects and underlying mechanisms of AMP dysregulation in AD progression.

## INTRODUCTION

Alzheimer's disease (AD) is a progressive neurodegenerative disorder that affects a growing proportion of the aging population. Patients with AD manifest with gradual decline of cognitive and functional abilities and shortened lifespan [1]. Due to the complex and multifactorial nature of AD, the etiology of which remains poorly understood, effective interventional means for prevention and treatment are lacking [2, 3]. There is growing recognition that the pathological mechanisms underlying AD do not only involve the aggregation of abnormal proteins, such as amyloid beta peptide (Aβ) and tau, but also include dysfunction of immune responses in the brain [4]. Since there is a lack of adaptive immune system in human brains under normal circumstances, impaired innate immune function has been proposed to be a key mechanism in the initiation and progression of AD [5]. Although the innate immune system has been considered a potential therapeutic target and has drawn substantial attention in biological and pharmaceutical studies, it is still disputed whether innate immunity is increased or decreased in AD [6, 7].

Animal models are indispensable tools to investigate pathological mechanisms and intervention strategies for AD. Over the past few decades, many studies have been conducted in *Drosophila melanogaster* to gain insight into the pathophysiological processes underpinning AD, identify potentially important genes and biomarkers, and screen new drug candidates. Besides the well-known advantages of using *Drosophila* as a model species [8], the host defense of the fruit fly rests entirely within its complex innate immune system, which makes it a desirable model for research on innate immunity in AD [9].

Aging is generally regarded as the most important risk factor for AD. However, the effects of aging on innate immunity in *Drosophila* have not been fully elucidated. Therefore, we performed a comprehensive data mining of the published expressional profiles [10–23] and experimental study at the transcriptomic level to analyze the expression profiles of innate immunity genes in wild-type (WT) and Aβ transgenic *Drosophila* model during aging. The transcriptional levels of major differentially expressed genes, Aβ deposition, and neuronal apoptosis in the head of both control and AD flies were also assessed to evaluate the effects of dysregulation of innate immunity on disease progression.

## RESULTS

### Transcriptomic data mining revealed a general upregulation of antimicrobial peptide (AMP) expression in the head of normal *D. melanogaster* during aging

To explore the expressive regularity of the innate immune system from a broad range of data, a total of 18 eligible experiments from GEO database comprising data of 52 young, 57 middle-aged, and 75 old healthy *Drosophila* head samples were obtained through data retrieval and selection of *Drosophila* head samples (Table 1). All expression profiles of innate immune genes were converted into occurrence of high expression. RD and OR values, which represented the difference in expression of innate immune genes, were calculated subsequently.

**Table 1:** Eligible datasets mined from GEO database.

**GEO accession**	**Sample**	**Age**	**Age group**
GSE122470	GSM3466957, GSM3466958, GSM3466959	3	Young
	GSM3466960, GSM3466961, GSM3466962	15	Mid-aged
	GSM3466963, GSM3466964, GSM3466965	30	Old
	GSM3466966, GSM3466967, GSM3466968	45	
GSE75216 [10]	GSM1945845, GSM1945846, GSM1945847, GSM1945855, GSM1945856	7	Young
	GSM1945843, GSM1945844, GSM1945853, GSM1945854	22	Mid-aged
GSE64108	GSM1564407, GSM1564408, GSM1564409, GSM1564410	21	Mid-aged
	GSM1564415, GSM1564417, GSM1564419, GSM1564421	35	Old
	GSM1564431, GSM1564432, GSM1564433, GSM1564434	49	Old
GSE38998 [11]	GSM1186462, GSM1186463	3	Young
	GSM953478, GSM953479	10	Mid-aged
GSE81100 [12]	GSM2143625, GSM2143626, GSM2143627, GSM2143628, GSM2143629, GSM2143630, GSM2143631, GSM2143632, GSM2143633, GSM2143634, GSM2143635, GSM2143636	5	Young
	GSM2143637, GSM2143638, GSM2143639, GSM2143640, GSM2143641, GSM2143642, GSM2143643, GSM2143644, GSM2143645, GSM2143646, GSM2143647, GSM2143648	55	Old
GSE110135 [13]	GSM2978238, GSM2978239, GSM2978240	3	Young
	GSM2978241, GSM2978242, GSM2978243	20	Mid-aged
GSE6430	GSM12770	3	Young
	GSM12772	47	Old
GSE97493 [14]	GSM2570129, GSM2570130, GSM2570131, GSM2570132, GSM2570133	3	Young
	GSM2570134, GSM2570135, GSM2570136, GSM2570137, GSM2570138, GSM2570159, GSM2570160, GSM2570161, GSM2570162, GSM2570163, GSM2570164	10	Mid-aged
	GSM2570149, GSM2570150, GSM2570151, GSM2570152, GSM2570153	30	Old
	GSM2570154, GSM2570155, GSM2570156, GSM2570157, GSM2570158	45	Old
GSE98554 [15]	GSM2599109, GSM2599110, GSM2599111	2	Young
	GSM2599112, GSM2599113, GSM2599114	25	Mid-aged
GSE48681 [16]	GSM1183416, GSM1183417, GSM1183418, GSM1183419	3	Young
	GSM1183420, GSM1183421, GSM1183422, GSM1183423	10	Mid-aged
	GSM1183424, GSM1183425, GSM1183426	20	
	GSM1183427, GSM1183428, GSM1183429, GSM1183430	56	Old
	GSM1183435, GSM1183436, GSM1183437, GSM1183438	68	
GSE25009 [17]	GSM614349, GSM614350, GSM614351	3	Young
	GSM614352, GSM614353, GSM614354	30	Old
	GSM614355, GSM614356, GSM614357	60	
GSE26246 [18]	GSM644354, GSM644355, GSM644356	0	Young
	GSM644357, GSM644358, GSM644359	2	
	GSM644360, GSM644361, GSM644362	14	Mid-aged
GSE26726 [19]	GSM658027, GSM658028, GSM658029, GSM658036, GSM658037, GSM658038, GSM658060, GSM658061, GSM658062	10	Mid-aged
	GSM658042, GSM658043, GSM658044, GSM658051, GSM658052, GSM658053, GSM658066, GSM658067, GSM658068	40	Old
GSE22440	GSM557543, GSM557544, GSM557545	10	Mid-aged
	GSM557546, GSM557547, GSM557548	40	Old
GSE21182 [20]	GSM530094	1	Young
	GSM530096	40	Old
GSE6314 [21]	GSM132562, GSM132563	15	Mid-aged
	GSM132564, GSM132565	20	
	GSM132566, GSM132567	30	Old
	GSM132568, GSM132569	45	
	GSM132570, GSM132571	60	
GSE826 [22]	GSM12770	3	Young
	GSM12772	47	Old
GSE37148 [23]	GSM912518, GSM912519, GSM912520	5	Young
	GSM912521, GSM912522, GSM912523	45	Old

**Figure 1:**
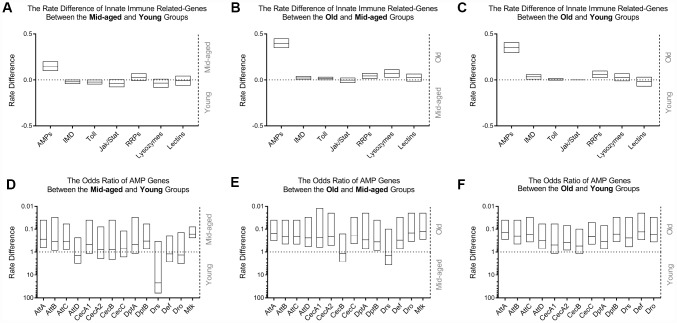
**Comparison of innate immunity gene expression variances in WT flies with age among GEO datasets.** The occurrence of high expression was introduced as a statistical parameter to compare the relative expression quantity of immune-related genes across age groups. The upper figures exhibit the rate differences among different classes of genes associated with innate immunity between the middle-aged and young (**A**), old and middle-aged (**B**), and old and young (**C**) groups, clearly demonstrating that AMPs had the most significant differential expression. The expression levels of other gene clusters were generally unchanged. Transcriptional differences in AMP genes were subsequently compared using meta-analysis. The odds ratio between the middle-aged and young (**D**), old and middle-aged (**E**), and old and young (**F**) groups, are shown in the lower graphs, indicating an increased expression of AMPs in the head of WT *Drosophila* during aging.

Among all categories of innate immunity relevant genes (Figure 1A–1C), only AMP genes exhibited a marked increase in gene expression with age. However, the degree of expression of other inducible and constitutive effector molecules in the host defense system of *Drosophila*, such as C-type lectins or lysozymes; pattern recognition receptors (PRRs), including peptidoglycan recognition proteins (PGRPs) and gram-negative binding proteins (GNBPs); major molecules involved in Toll, immune deficiency (IMD), and Janus kinase - signal transducer and activator of transcription (JAK-STAT) pathways, essentially remained unchanged. Meta-analysis (Figure 1D–1F, Supplementary Figure 1) further demonstrated that with the exception of drosomycin (*Drs)*, among 14 major AMP genes, including attacin (*Att*) *A*, *B*, *C* and *D*; cecropin (*Cec*) *A1*, *A2*, *B* and *C*; diptericin (*Dpt*) *A* and *B*; *Drs*; defensin (*Def*); drosocin (*Dro*); and metchnikowin (*Mtk*); there was overexpression of all other genes between at least one younger group with that of at least one older group. The data mining results suggested that the upregulation of AMPs in the head of healthy aging *Drosophila* could be one of the most important changes in the innate immune system with age.

### Age-associated overexpression of AMP genes in normal *Drosophila* was observed in RNA-Seq

**Figure 2:**
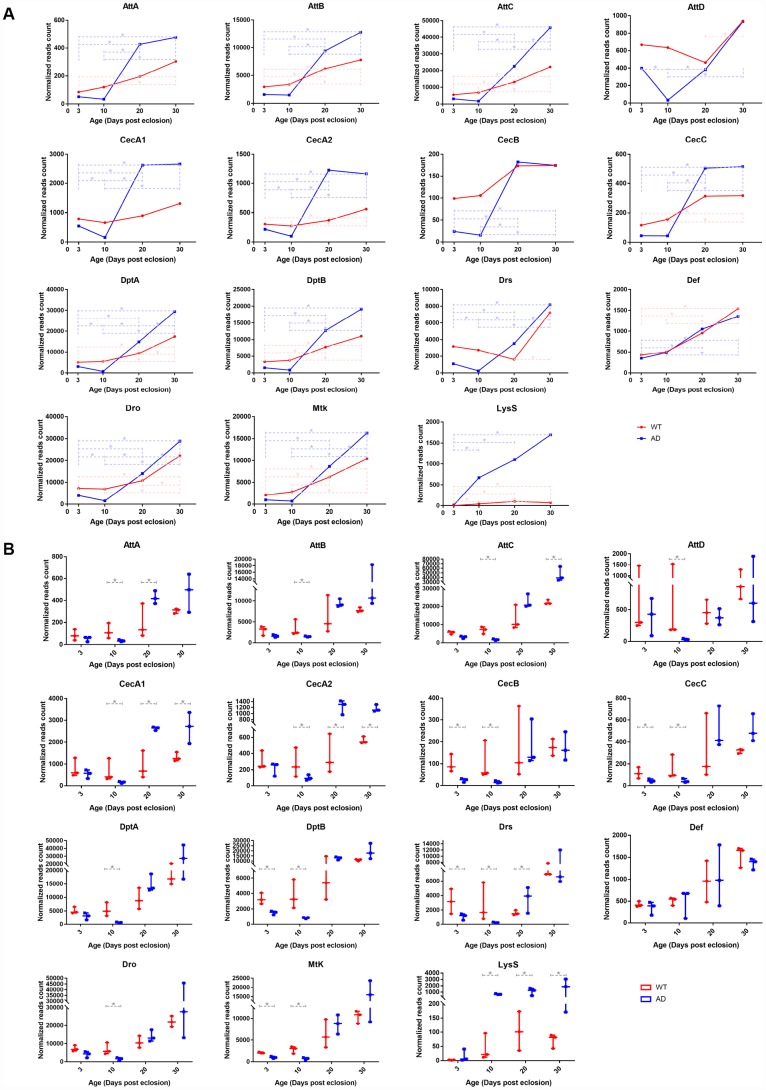
**RNA-seq analysis of differentially expressed genes associated with the innate immune system in control and AD *Drosophila* model.** There were significant differences in transcriptional levels of AMPs and *LysS* between the two groups. The line chart (**A**) illustrates different expression patterns of differentially expressed immune-related genes between WT (red, round dots) and AD (blue, square dots) groups during aging. The box plot (**B**) presents differences in expression therein between the normal (red, left) and disease (blue, right) groups at each time point (3-, 10-, 20-, 30-days post eclosion).

To validate the gene expression variation of the innate immune system with age, whole transcriptome profiles of normal *Drosophila* heads at 3, 10, 20, and 30 days post eclosion were analyzed by high throughput RNA sequencing. The raw data had been deposited to the GEO database with an accession number of GSE109489. The relative quantity of gene expression and the significance value between groups were calculated by DESeq2 software. The results revealed that 12 out of 14 AMP genes, including *AttA*, *AttB*, *AttC*, *AttD*, *CecA2*, *CecC*, *DptA*, *DptB*, *Drs*, *Def*, *Dro*, and *Mtk*, had upregulated expression in at least one older group compared to that of at least one younger group (Figure 2B). Despite of slight variations on individual genes, the expression levels of these AMP genes generally presented a gradual tendency to increase with age, consistent with results from the big data analysis (Figure 2A). Lysozyme (*Lys*) *S*, and three PGRP genes, including *-LC*, *-SA*, and *-SD*, were overexpressed in at least one pair of age groups. However, there was no significantly different expression among other AMP-related genes.

### High throughput sequencing indicated differential AMP expression pattern in normal and AD flies

Transcriptomic profiling of the *Drosophila* model of AD at the time points corresponding to those of the WT group was performed using RNA-Seq. Raw transcriptomic data was deposited to the same database with an identical accession number. Time series analysis illustrated that the expression of most AMPs declined to the lowest on day 10, elevated sharply on day 20, and continuously increased or remained unchanged on day 30. The expression levels of AMP genes displayed a rough trend of initial decrease followed by an increase, which was distinct to that of healthy controls (Figure 2A). The most significant differences in expression between WT and AD groups appeared on day 10, when 13 out of 14 AMPs, including *AttA*, *AttB*, *AttC*, *AttD*, *CecA1, CecA2*, *CecB, CecC*, *DptA*, *DptB*, *Drs*, *Def*, *Dro*, and *Mtk*, showed a remarkable decline in the AD group, with log2 fold changes of -1.816, -1.182, -2.011, -4.250, -2.065, -1.455, -2.735, -1.813, -3.011, -2.184, -3.525, -2.173, and -1.975, respectively (Figure 2B). mRNA levels of *LysS*, *PGRP-LC*, *-SA*, and *-SD* genes presented a rising trend with age in the AD group, but there were no significant differences among PGRP genes when compared with those of the WT group. However, the expression of *LysS* exhibited a comparable increase in *Drosophila* with AD. The log2-fold change values of *LysS* between the WT and AD groups at day 3, 10, 20, and 30 were 2.891, 3.929, 3.412, and 4.576, respectively (Figure 2).

### Validation of AMP gene expression profiles by quantitative real-time PCR (qPCR)

**Figure 3:**
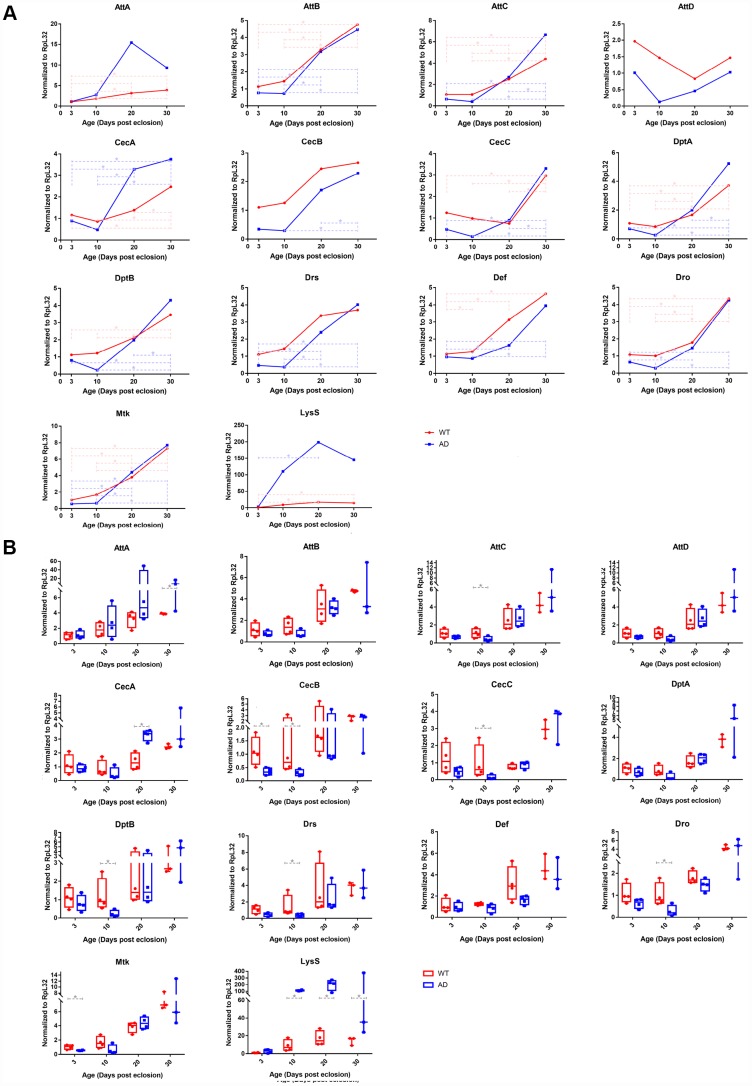
**Quantitative PCR validation of differentially expressed immune-related genes in control and Aβ transgenic flies.** The results confirmed age-associated alterations in expression trends and transcriptional regulatory levels among the AMP and *LysS* genes between healthy control and disease model flies. The line chart (**A**) displays the time series (3-, 10-, 20-, and 30-days post eclosion) gene expression in the head tissue of WT (red, round dots) and AD (blue, square dots) flies. The box plot (**B**) exhibits the comparison of mRNA levels between normal (red, left) and disease (blue, right) model flies among the age groups.

Transcript levels of key differentially expressed innate immunity genes were investigated using RNA-Seq, including 14 AMP genes and *LysS*, and were validated using qPCR assays. The qPCR result generally recapitulated RNA-Seq data. Expression trends of the aforementioned genes in different age groups were generally consistent with those revealed by transcriptomic analysis (Figure 3A). Quantitative analysis confirmed that expression levels of nearly all AMP genes declined to some extent in the AD group at day 3 and 10. Among these, six AMPs, including *AttC*, *CecB*, *CecC*, *DptB*, *Drs*, and *Dro*, had the most significant differential expression, with log2-fold changes of -1.344, -2.097, -2.750, -2.398, -1.973, and -1.791, respectively. After day 10, AMP expression levels in the AD group rapidly increased. The expression of most AMP genes remained at similar levels to those of the control group, of which *CecA* and *AttA* were found elevated on day 20 and 30 with log2-fold changes of 1.250 and 1.255, respectively (Figure 3B). There was a continuously increasing trend in the mRNA level of *LysS* from day 3 to 30, which was markedly increased relative to control levels, with log2-fold change values of 1.413, 3.636, 3.555, and 3.331, respectively (Figure 3).

### ELISA suggested that dysregulation of AMP expression was positively correlated with Aβ_42_ concentration but not neuronal apoptosis in AD flies

**Figure 4:**
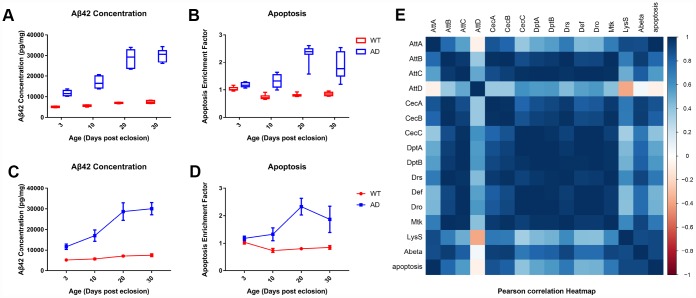
**Quantitative determination of Aβ_42_ and apoptosis levels in brain tissue of control and AD model flies.** Aβ_42_ concentration and apoptotic DNA fragmentation representing the extent of apoptosis was examined with ELISA. Compared to the control group (left), which had low levels of Aβ_42_ concentration (**A**, left) and cell apoptosis (**B**, left), the AD group had an increased concentration of Aβ (**A**, right) and apoptotic DNA fragments (**B**, right). The trends of Aβ_42_ production (**C**) and neuronal apoptosis (**D**) in the WT (round dots) and AD (square dots) groups are displayed in the lower figures. Subsequent correlation analysis further indicated a potential relationship between AMP expression, Aβ_42_ production, and neuronal apoptosis.

The levels of Aβ_42_ and neuronal apoptosis were determined as described previously using respective ELISA kits. ELISA revealed significantly increased Aβ_42_ concentration and apoptotic DNA fragmentation within the disease group, indicating abnormal Aβ aggregation and neuronal apoptosis in *Drosophila* with AD (Figure 4A and 4B). Time series analysis showed that the degree of Aβ burden and apoptosis in the head of WT flies remained at low levels over time, whereas that of the AD group presented an obvious upward trend from day 3 to30. The degree of Aβ and apoptosis were most significantly increased on day 20 (Figure 4C and 4D).

Pearson's correlation coefficient test (Figure 4E) revealed a significant positive correlation between the generation of human Aβ_42_ peptide and programmed cell death in the head of the *Drosophila* model. No significant correlation was detected between AMP genes or *LysS* expression and apoptosis. Nevertheless, significant correlations between abnormal Aβ concentration and aberrant expression of *AttB*, *AttC*, *CecA*, *Drs*, *Mtk*, and *LysS* genes were detected.

## DISCUSSION

*Drosophila* has proven to be an excellent model system for studies of aging and age-related neurodegenerative diseases, not only due to its high genetic conservation to humans, but also due to its short lifespan and simple central nervous system (CNS) anatomy and physiology. In humans, aging and neurodegenerative diseases are accompanied with altered immunity [24]. Although there is a lack of adaptive immunity in *Drosophila*, immune defense in the human brain under normal circumstances also relies entirely on the innate immune system [5]. The innate immune systems of humans and *Drosophila* are highly analogous: they are activated by PGRPs and GNBPs; transcriptionally regulated through Toll, IMD, and JAK-STAT signaling pathways; and function by expressing effector molecules through NF-κB transcription factors [25, 26].

Innate immunity is the first line defender of the host based on efficient response mechanisms and potent immune molecules that are expressed, such as AMPs, lysozymes, and lectins. The innate immune system has been proposed to be upregulated during aging to withstand growing susceptibility to infections [27]. Conversely, overexpression of antimicrobial peptide genes could contribute to age-related diseases through cytotoxic effects [28]. Although the interplay between immunity and aging in *Drosophila* is well established, despite that the mechanism that underlie such reciprocity remain unknown, there is a lack of literature specific to the head tissue. In this study, gradually increasing levels of AMP expression in the head of healthy aging *Drosophila* were observed by mining transcriptome sequencing data from the GEO database, which were subsequently verified by RNA-seq and qPCR analyses, consistent with the available literature [29–31]. However, there were no significant differences in expression of major genes of Toll, IMD, or JAK-STAT signaling pathways, indicating that these classical immune relevant pathways may not be involved in the overexpression of AMP genes.

Prolonged inflammation is associated with the progression of AD in humans, which results from the accumulation of aberrant Aβ aggregates. Activated immunity in the CNS has been suggested to be responsible for the onset of neurodegeneration in *Drosophila* [32]. Experimental evidence has illustrated that overexpression of a particular AMP gene in the CNS of WT *Drosophila* was sufficient to induce pathogenesis of neurodegeneration and shortened lifespan. The pathological mechanism was attributed to the cytotoxicity of AMPs on neurons and glia in the CNS [28, 33]. Neuron loss, behavioral impairment, and shortened lifespan are the most characteristic pathological changes of *Drosophila* with AD. Therefore, the expression of AMP genes were deduced to be upregulated in the AD *Drosophila* model. In this study, *LysS*, which was the only differentially expressed disease-associated immune response gene other than AMPs, was strongly overexpressed in the AD group. Overexpression of lysozymes inhibits Aβ aggregation and cell apoptosis, making it a potential target for the diagnosis and treatment of AD [34]. Interestingly, to our surprise, the expression of AMPs were downregulated in the early stage of adult AD flies based on our results, especially on day 10, indicating that overexpression of AMPs is not an indispensable condition for the pathogenesis of AD. In contrast, the downregulation of AMPs occurred prior to commencement of substantial Aβ deposition and neuronal apoptosis on day 20, suggesting that the suppressed expression of AMPs may initiate the development of AD and may be a novel hallmark for early diagnosis of AD. Nevertheless, the expression levels of most AMP genes were generally increased compared to those of controls at the later stage of AD. Under such circumstances, AMPs may exert their cytotoxic effects on CNS cells and contribute to disease progression.

Available transcriptomic profiles from the GEO database include three studies (Supplementary Figure 2) on *Drosophila* models of AD (GSE48681), amyotrophic lateral sclerosis (ALS, GSE37148) and Parkinson’s disease (PD, GSE74247) [16, 23, 35]. The experiment revealed a generally decreased expression of AMPs in the neurodegenerative disease group compared with the relative controls at early ages, although only a few of the differences among individual AMPs were statistically significant. Low levels of AMP gene expression may be a common phenomenon in the early stages of neurodegenerative diseases. However, the time points set after 20 days in the AD study were not matched by age between the disease and control groups, so it is difficult to determine the variation in AMP expression in *Drosophila* with advanced AD, although generally elevated AMP mRNA levels could be observed at the late stage of ALS. A microarray assay of AD also demonstrated that AMP genes were downregulated when the mortality of *Drosophila* with AD started to increase. Our study further illustrated that there was no significant association between AMP expression and neuronal apoptosis, according to correlation coefficient analysis. Therefore, decreased AMP expression is unlikely to be directly responsible for neuronal loss or mortality in *Drosophila* models of AD. Notably, correlation analysis pointed to trends towards positive correlations between expression of several antimicrobials and neurotoxic Aβ_42_ concentration. These findings suggest that downregulation of AMP expression presumptively lead to Aβ deposition, resulting in neuronal apoptosis and mortality.

AMP gene expression relies predominantly on the Toll and IMD signaling pathways. The JAK-STAT pathway is also involved in the regulation of innate immune responses [36]. Activation of Toll and IMD pathways leads to AMP expression, while absence of both pathways results in loss of AMP production [37]. It is noteworthy that Aβ is increasingly being recognized as an AMP that protects the host against pathogenic microorganism infection [38]. Therefore, downregulated expression of Aβ peptide could be mediated by negative feedback mechanisms. The expression of Aβ in the transgenic AD *Drosophila* model that was used in this study was transcriptionally regulated by the GAL4 protein, hence a competitive transcription factor binding mechanism could not be responsible for the low expression of AMP genes. Moreover, in this study, increasing PGRP expression with age was detected in both the AD and WT groups. However, no significant differences in expression among the major genes of the Toll, IMD, or JAK-STAT pathways between the disease and control groups were detected in either *in silico* or experimental analyses; the mechanisms involved in aberrant AMP expression therefore remain unclear.

In conclusions, the innate immune systems of AD and WT *Drosophila* were systematically analyzed using an integrative strategy of transcriptomics and experimental validation in this study. The degree of Aβ production and neuronal apoptosis was also investigated. The expression of AMPs in the WT group increased gradually with time, but the increasing trend of AMP expression was disrupted in the AD group, which exhibited an initial downward trend followed by an upward trend during aging. The occurrence of disordered AMP expression, massive aberrant Aβ aggregates, and significant neuronal apoptosis appeared in sequential order, and correlation analysis further indicated a possible causal relationship among the variables. In conclusion, our study revealed dysregulation of AMP expression in an AD *Drosophila* model with age, distinct from normal aging. Disordered AMP expression may contribute to AD progression by inducing Aβ deposition. However, the physiological and pathological mechanisms of aberrant AMP regulation and the effects on AD and healthy aging are yet to be discovered.

## MATERIALS AND METHODS

### Analysis of innate immune gene expression in the head of normal *D. melanogaster* during aging based on data mining strategies

To analyze the gene expression of innate immunity in *Drosophila* with age, transcriptome profiles, including microarray and high-throughput sequencing data, were retrieved from the GEO database by organism-specific keyword searching using terms of aging, age, and “time course.” Eligible experiments had to include head tissue of normal *D. melanogaster,* which were reared under routine culture conditions without any stimulation and contain at least two age groups (young: < 10 days; middle-aged: 10-29 days; old: ≥ 30 days) in a single experiment. The gene expression value was extracted from the original published datasets. The log2-fold change of gene expression relative to control was calculated. Values greater than 1 (2 folds) were considered significant. We compared the statistical parameters of differential expression obtained from individual datasets instead of comparing gene expression signals between experiments to avoid the difficulties in comparing data from different conditions. Each sample from an individual dataset was compared with other age groups. If the log2-fold change exceeded the cutoff threshold, it was marked as one occurrence of high expression. The number of occurrences (*k*) and number of samples in a single experiment (*n*) were counted separately.

For comparison of a certain class of genes, the frequency of high expression genes (response rate, P) was calculated (P = *k* / *n*), and the overall expression differences were presented as rate difference (RD) = response rate of the older group (Po) - response rate of the younger group (Py). For comparison of the expression of an individual gene among different age groups, the occurrence of high expression genes and total events were counted separately. The analysis was performed using Review Manager Version 5.0. The odds ratios (OR) were the principal measurements of the effects and were presented with 95% confidence intervals (CI). Differences with *p* < 0.05 were considered statistically significant.

### *Drosophila* stocks

The *Drosophila* model of AD that expresses human Aβ_42_ peptide in the brain was constructed using a cross of the w^1118^ genetic background UAS-Aβ_42_ flies driven by elav-GAL_4_^c155^ line, while WT (w^1118^) flies which also outcrossed with the elav-GAL_4_^c155^ line were used as controls [39]. All flies were reared at 23°C and 42% relative humidity, and fed with standard corn meal food under 12 h/12 h light/dark cycles. Head tissues from 3-, 10-, 20, and 30-day-old male WT and AD *Drosophila* were collected for further experimentation.

### RNA isolation and gene expression analysis

An approximate 200 fly heads for each of triplicates for each age group were collected, and total RNAs were extracted with Trizol reagent (Thermo Fisher, USA), from which mRNAs were purified by poly-T oligo-attached magnetic beads. Libraries were constructed for sequencing using a Superscript Double-Stranded cDNA Synthesis kit (Thermo Fisher, USA) according to manufacturer's specifications.

The prepared library was sequenced on the HiSeq X Ten platform (Illumina, USA) based on a 2 × 150 bp paired-end (PE150) sequencing protocol. The raw sequencing data were processed with SOAPnuke 1.5.2 with parameters of -l 15 -q 0.2 -n 0.05 -i, to remove adaptor sequences, poly-N reads, and low quality reads [40]. The obtained clean data were mapped to *Drosophila melanogaster* whole genome dmel_r6.11 using HISAT 2.0.4 with parameters of --phred64 --sensitive --no-discordant --no-mixed -I 1 -X 1000 [41, 42]. The relative quantities of gene expression were calculated using RSEM software.

The levels of differentially expressed innate immunity genes were further elucidated through standard qPCR experiment subsequently. Specific primers used in qPCR analysis are listed as Supplementary Table 1. The assays were performed in triplicate on ABI ViiA 7 Real-time PCR system (Applied Biosystems, USA) using QuantiTect SYBR Green PCR kit (Qiagen, Germany) as the fluorescent reporter. Relative expression was estimated with ribosomal protein L32 (*RpL32*) as the reference gene using the 2^−∆∆Ct^ method.

### Protein extraction and ELISA assays

Aβ expression in the brains of *Drosophila* was examined using a High Sensitivity Human Amyloid β42 ELISA kit (Merck Millipore, USA) according to the manufacturer’s instruction. Briefly, 20 fly heads of each of three replicates for each age group were homogenized then diluted in Standard and Sample Diluent followed by centrifugal purification. The supernatant was transferred into a capture-antibody-coated ELISA plate for overnight incubation at 4°C. After careful rinsing with washing solution, the plate was incubated with the biotinylated detection antibody to form an antibody-amyloid-antibody-complex, which was visualized using the streptavidin-HRP method and measured at 450 nm with a microplate reader (Pulang, China).

Neuronal apoptosis in *Drosophila* was determined with a Cell Death Detection ELISA Plus kit (Roche, Switzerland). In brief, homogenate samples were prepared using the same method described above. The supernatant containing cytoplasmic histone-associated DNA fragments was added to a streptavidin-coated microplate and incubated with a mixture of anti-histone (biotin-labeled) and anti-DNA (peroxidase-conjugated) antibodies that included in the ELISA kit. The DNA-histone-complex was colored with the ABTS Substrate, then measured at 405 nm with a microplate reader (Pulang, China).

### Statistical analysis

Transcriptomic analysis for *in silico* data was conducted using the DESeq2 package of R software [43]. Transcripts with absolute values of log2-fold change greater than 1 and false discovery rates less than 5% were considered to be significantly differently expressed. Statistical analyses for ELISA and qPCR studies were performed using SPSS Statistics 19.0 software and presented as mean ± SD. Dynamic changes in gene expression were statistically analyzed using one-way analysis of variance (ANOVA), with the Bonferroni post-hoc multiple comparison tests. Significance of qPCR validation among age groups was examined using the Mann-Whitney *U* test. Correlations between differential gene expression, Aβ_42_ concentration, and neuronal apoptosis were examined using Pearson's correlation analysis. Results were plotted using the corrplot package of R software. A *p*-value <0.05 was considered statistically significant.

## Supplementary Material

Supplementary Figures

Supplementary Table 1
