# Supplementary material for: Dysregulation of antimicrobial peptide expression distinguishes Alzheimer’s disease from normal aging
Source: Aging (Albany NY). 2020 Jan 6;12(1):690–706. doi: 10.18632/aging.102650 (PMC6977672; doi:10.18632/aging.102650)
Supplement: Supplementary Figures [file aging-12-102650-s002..pdf]

SUPPLEMENTARY FIGURES

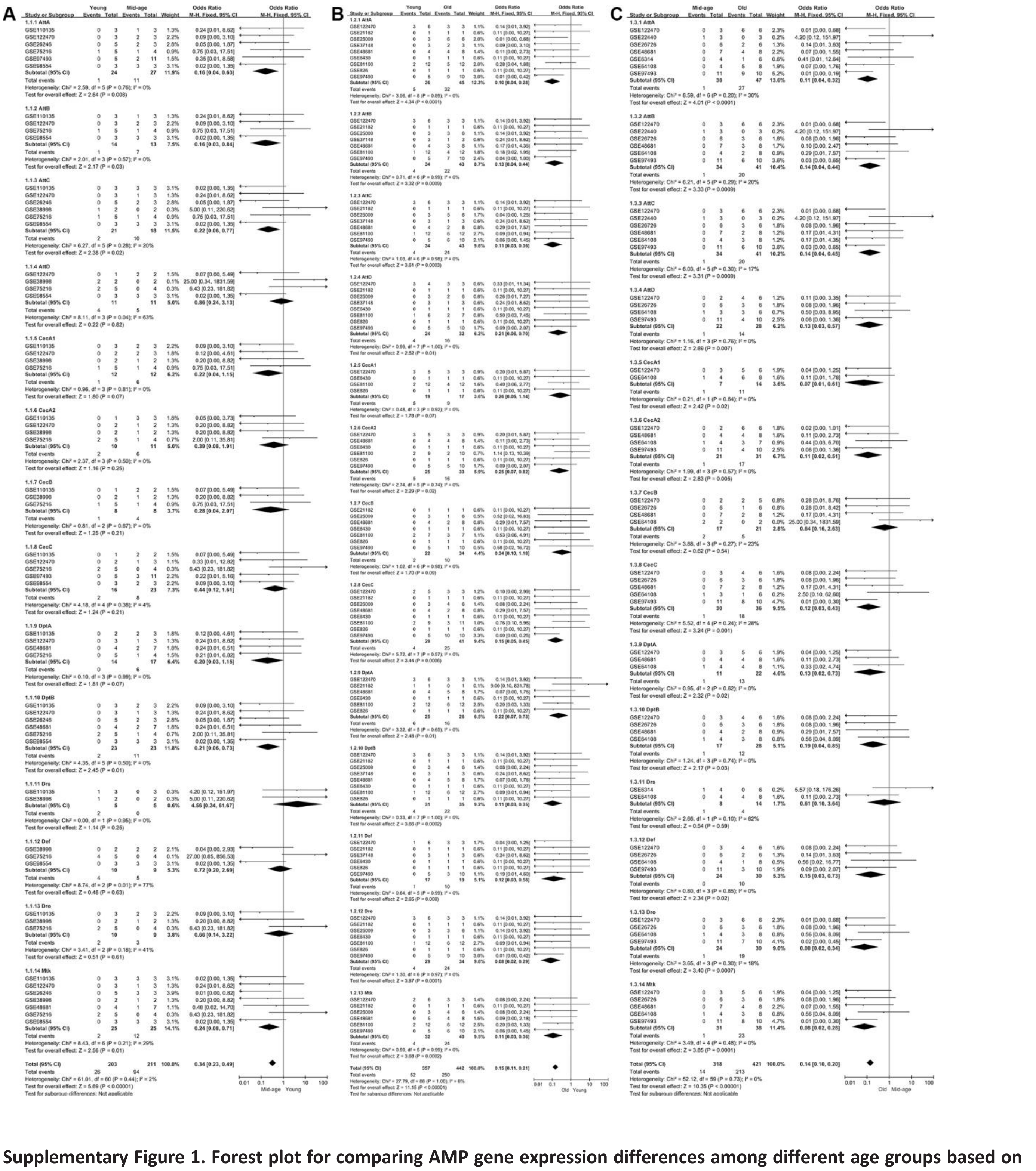

Supplementary Figure 1. Forest plot for comparing AMP gene expression differences among different age groups based on the data mined from the GEO database. The random and fixed effects model (Mantel-Haenszel method) was used in the meta-analysis. Comparison between the middle-aged and young (**A**), old and middle-aged (**B**), and old and young (**C**) groups all demonstrated increased expression among AMP genes in older groups, suggesting that there was a general rising trend in AMP expression during aging.

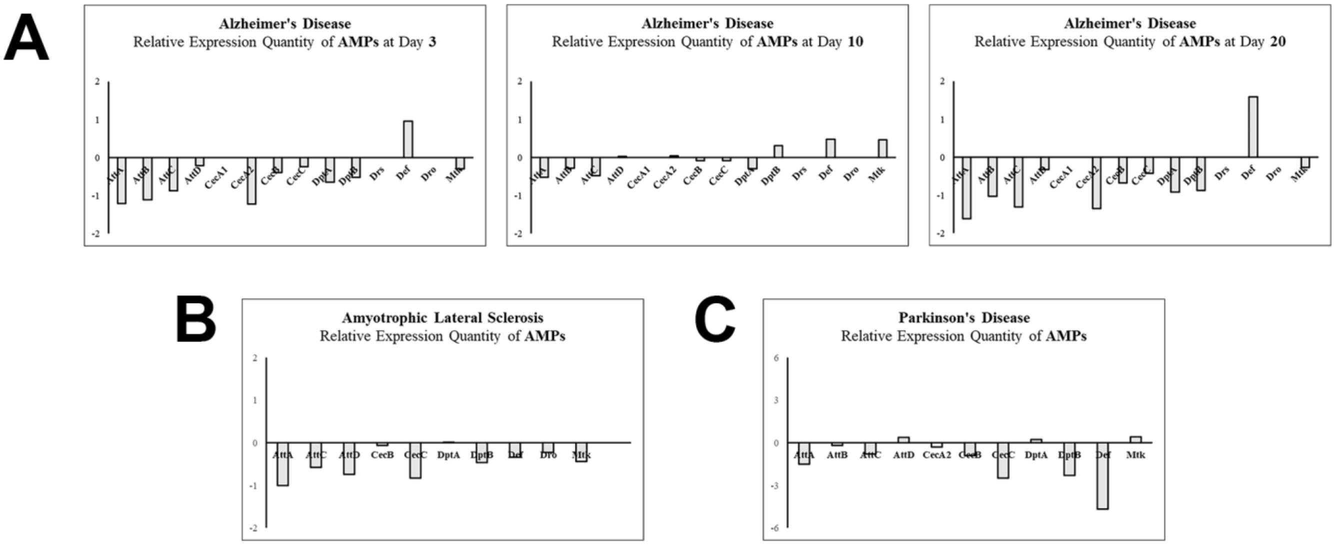

Supplementary Figure 2. Comparison of AMP gene expression between a *Drosophila* model of neurodegenerative disease and healthy controls by data mining. Bioinformatics analysis illustrated that the transcriptional levels of AMP genes exhibited general reductions in flies with AD (**A**), ALS (**B**), and PD (**C**) when compared to those of relative controls, suggesting that the downregulation of AMP genes may be a common phenomenon in neurodegenerative diseases.
