# Supplementary material for: Dysregulation of antimicrobial peptide expression distinguishes Alzheimer’s disease from normal aging
Source: Aging (Albany NY). 2020 Jan 6;12(1):690–706. doi: 10.18632/aging.102650 (PMC6977672; doi:10.18632/aging.102650)
Supplement: Supplementary Table 1 [file aging-12-102650-s001..pdf]

SUPPLEMENTARY TABLE

Supplementary Table 1. Primers used for quantitative real-time PCR (qPCR) analysis.

Gene	Forward (5′ to 3′)	Reverse (5′ to 3′)
AttA	ATCGCCCAATCGTGCTACTAC	ATGATGAGATAGACCCAGGCC
AttB	TCTCTGGTCATCGCCCAATC	CCAGCACCAAAGTTTGGCTT
AttC	TCATGGAGCTACCCTGACGC	AGCCTTGTGTTGCGATCCTG
AttD	AACGCCAATGGTCATGCACT	TCAGAGCGGCGTTATTGCTC
CecA	TTCGTCGCTCTCATTCTGGC	ATCCCGAGTGTGCTGACCAA
CecB	CACTCATCCTGGCCATCAGC	CGATTCCGAGGACCTGGATT
CecC	CTCATCCTGGCCATCAGCAT	CGCAATTCCCAGTCCTTGAA
DptA	TTTTGGCTTTGCAGTCCAGG	GTCCTCCCAAGTGCTGTCCA
DptB	CCCTATCCTGATCCCCGAGA	CCATTCAATTGGAACTGGCG
Drs	TCTTCGCTGTCCTGATGCTG	AGGTCTCGTTGTCCCAGACG
Def	AGGCTCAGCCAGTTTCCGAT	AGTAGGTCGCATGTGGCTCG
Dro	CTGCTGCTTGCTTGCGTTTT	GTGATCCTCGATGGCCAGTG
Mtk	CACGGCTACATCAGTGCTGG	AATTGGACCCGGTCTTGGTT
LysS	CGACGGACGCTTCTCCTACA	TGCTGGCTGAGGACCTTCTG
RpL32	GCCCAACATCGGTTACGGAT	TGCATGAGCAGGACCTCCAG
